# Cost-effectiveness of first-line versus delayed use of combination dapagliflozin and metformin in patients with type 2 diabetes

**DOI:** 10.1038/s41598-019-40191-8

**Published:** 2019-03-01

**Authors:** Ken Lee Chin, Richard Ofori-Asenso, Si Si, Thomas R. Hird, Dianna J. Magliano, Sophia Zoungas, Danny Liew

**Affiliations:** 1 CCRE Therapeutics, Department of Epidemiology and Preventive Medicine, Monash University, Melbourne, Australia; 2 Melbourne Medical School, The University of Melbourne, Parkville, Australia; 3 Baker Heart and Diabetes Institute, Melbourne, Australia; 4 Department of Epidemiology and Preventive Medicine, School of Public Health and Preventive Medicine, Monash University, Melbourne, Australia; 5 The George Institute for Global Health, Sydney, Australia

## Abstract

The present study sought to evaluate the cost-effectiveness of first-line (immediate) versus delayed use of combination dapagliflozin and metformin in patients with type 2 diabetes, from the perspective of the Australian healthcare system. We developed a Markov model to simulate the progress of subjects with type 2 diabetes. Decision analysis was applied to assess the cost-effectiveness of first-line combination dapagliflozin and metformin versus first-line metformin monotherapy followed by gradual addition of dapagliflozin over time. Transition probabilities, costs (in Australian dollars) and utility data were derived from published sources. All costs, years of life lived and quality adjusted life years (QALYs) lived were discounted at an annual rate of 5%. Over a 20-year model period, first-line use of combination dapagliflozin and metformin was predicted to reduce the onset of hospitalisation of heart failure, cardiovascular deaths and all cause deaths by 5.5%, 57.6% and 29.6%, respectively. An additional 2.5 years of life (discounted) and 1.9 QALYs (discounted) would be gained per patient, at a cost of AUD $23,367 (discounted) per person. These figures equated to AUD $9,535 per years of life saved (YoLS) and AUD $12,477 per QALYs saved. Sensitivity analyses indicated the results to be robust. Compared to first-line metformin monotherapy followed by gradual addition of dapagliflozin, first-line use of combination dapagliflozin and metformin is likely to be a cost-effective approach to the management of Australians with type 2 diabetes mellitus.

## Introduction

A rising prevalence of overweight/obesity and lack of physical activity have given rise to the epidemic of type 2 diabetes mellitus (T2DM) worldwide. Nearly 440 million people are projected to have T2DM by 2030, with more than 80% living in less developed countries^1^. Patients with T2DM are at 2- to 4-fold increased risk of heart failure and cardiovascular death compared with those without diabetes, even in the absence of ischaemic heart disease^2,3^.

Studies suggest that the mean HbA1c at initiation of oral antihyperglycaemic therapy is about 9%^4–6^. Globally, the proportion of treated patients not at optimal HbA1c target (≤7%) is between 35% and 87%, and the mean HbA1c level at the time of treatment intensification is between 8.7% to 9.7%^7^. Other than lifestyle management, contemporary guidelines recommend that dual therapy is initiated when HbA1c is ≥9% at diagnosis or if target HbA1c is not achieved after three months of monotherapy with metformin^8–11^. However, data from prospective observational studies suggest that there are often delays of three years or longer in intensifying glucose-lowering therapy among patients with poor control of HbA1c who need treatment escalation^7^.

Recent years have witnessed the emergence of evidence from phase III randomised clinical trials of the direct cardiovascular benefits of sodium-glucose-cotransporter 2 (SGLT2) inhibitors among patients with T2DM: Empagliflozin Cardiovascular Outcome Event Trial in Type 2 Diabetes Mellitus Patients - Removing Excess Glucose (EMPA-REG OUTCOME) study; and the Canagliflozin Cardiovascular Assessment Study (CANVAS) study^12,13^. Data from large observational studies have also suggested benefits. The ‘Comparative Effectiveness of Cardiovascular Outcomes in New Users of Sodium-Glucose Cotransporter-2 Inhibitors Nordic’ (CVD-REAL Nordic) study, which was sponsored by AstraZeneca, found that patients newly treated with any SGLT2 inhibitor had lower risks of cardiovascular mortality (hazard ratio [HR], 0.53; 95% confidence interval [CI], 0.40 to 0.71], major adverse cardiovascular events (HR, 0.78; 95% CI, 0.69 to 0.87) and hospitalisations for heart failure (HR, 0.70; 95% CI, 0.61 to 0.81) compared to patients not initiated on SGLT2 inhibitors^14^. In this study, propensity score-matched patients had a mean age of 61 years, 40% were women, and the baseline prevalence of cardiovascular disease (CVD) was 25%. Of note, mean follow-up was 0.9 years and 94% of the total SGLT2 inhibitor exposure time was use of dapagliflozin.

Recently (and subsequent to the submission of our original manuscript to the journal), results from the Dapagliflozin Effect on Cardiovascular Events–Thrombolysis in Myocardial Infarction 58 (DECLARE–TIMI 58) trial and ‘CVD-REAL 2’ study were published^15,16^. In DECLARE-TIMI 58, dapagliflozin significantly reduced hospitalisation for heart failure (HF) (HR, 0.73; 95% CI, 0.61 to 0.88) and renal events (HR, 0.53; 95% CI, 0.43 to 0.66)^15^. The CVD-REAL 2 study^16^, which examined association between the use of SGLT2 inhibitors and a broad range of cardiovascular outcomes in the Asia Pacific, Israel and Canada, had reported similar findings compared to CVD-REAL Nordic. Specifically, use of SGLT2 inhibitors (75% of which was dapagliflozin) versus oral glucose lowering drugs was associated with a lower risk of death (HR, 0.51; 95% CI, 0.37 to 0.70), hospitalisation for HF (HR, 0.64; 95% CI, 0.50 to 0.82), MI (HR, 0.81; 95% CI, 0.74 to 0.88), and stroke (HR, 0.68; 95% CI, 0.55 to 0.84). In addition, subsequent meta-analysis of EMPA-REG OUTCOME, CANVAS and DECLARE-TIMI 58 indicated that the cardio-renal benefits remained consistent between the trials when analysed within similar patient subgroups, despite significant heterogeneity in study design and patient characteristics^17^.

Whether or not up-front use of combination SGLT2 inhibitors and metformin would be more cost-effective than later use of the combination is unclear. Early initiation of combination SGLT2 inhibitor and metformin may allow patients to achieve HbA1c targets more rapidly, leading to a shorter duration of inadequate glycaemic control, as well as weight loss and better blood pressure control. In addition, there may be direct cardiovascular benefits, as suggested by EMPA-REG OUTCOME, CANVAS, DECLARE-TIMI 58, CVD-REAL Nordic and CVD-REAL 2^12–16^. At present, metformin monotherapy is usual first-line treatment, while SGLT2 inhibitors are recommended for second- and third-line use^8,9,11^. Dapagliflozin, which was the first marketed in class and remains most commonly prescribed SGLT2 inhibitor in Australia (80% of the market)^16^, is subsidised under the Australian Pharmaceutical Benefits Scheme (PBS) for use with metformin or sulphonylureas, as triple oral therapy, and for use with insulin^11^. In the present study, we sought to estimate the effectiveness and cost-effectiveness of first-line compared with delayed use of combination dapagliflozin and metformin, from the perspective of the Australian healthcare system. Our analysis focused on patients with T2DM who were eligible for metformin monotherapy, as recommended by contemporary guidelines.

## Research Design and Methods

### Prescribing of metformin and SGLT2 inhibitors

The Pharmaceutical Benefits Scheme (PBS) is part of Australia’s universal health coverage mechanism and makes prescription medications available at subsidised costs to Australians^18^. To get insight into the prescribing of metformin and SGLT2 inhibitors, data were extracted from the ‘10% PBS dataset’, which includes data on a non-identifiable randomly-selected 10% sample of PBS patients. The dataset includes information on dispensed medications (item number, name, quantity and date of dispensing), demographic information (sex, year of birth, and year of death, if applicable), concessional status, prescriber information (identification number and specialty area), and the state or territory of the dispensing pharmacy. Data between 1 January 2013 and 31 December 2016 were analysed to determine the time taken to add a SGLT2 inhibitor in patients who were initiated on metformin monotherapy. Thereafter, a trend line over a period of 20 years was fit based on the annualised rate of addition of dapagliflozin to metformin. Patients who were dispensed with medications belonging to the ATC code A10 were deemed to have diabetes, as has been done in previous studies^19,20^.

### Model and modelled population

**Figure 1:**
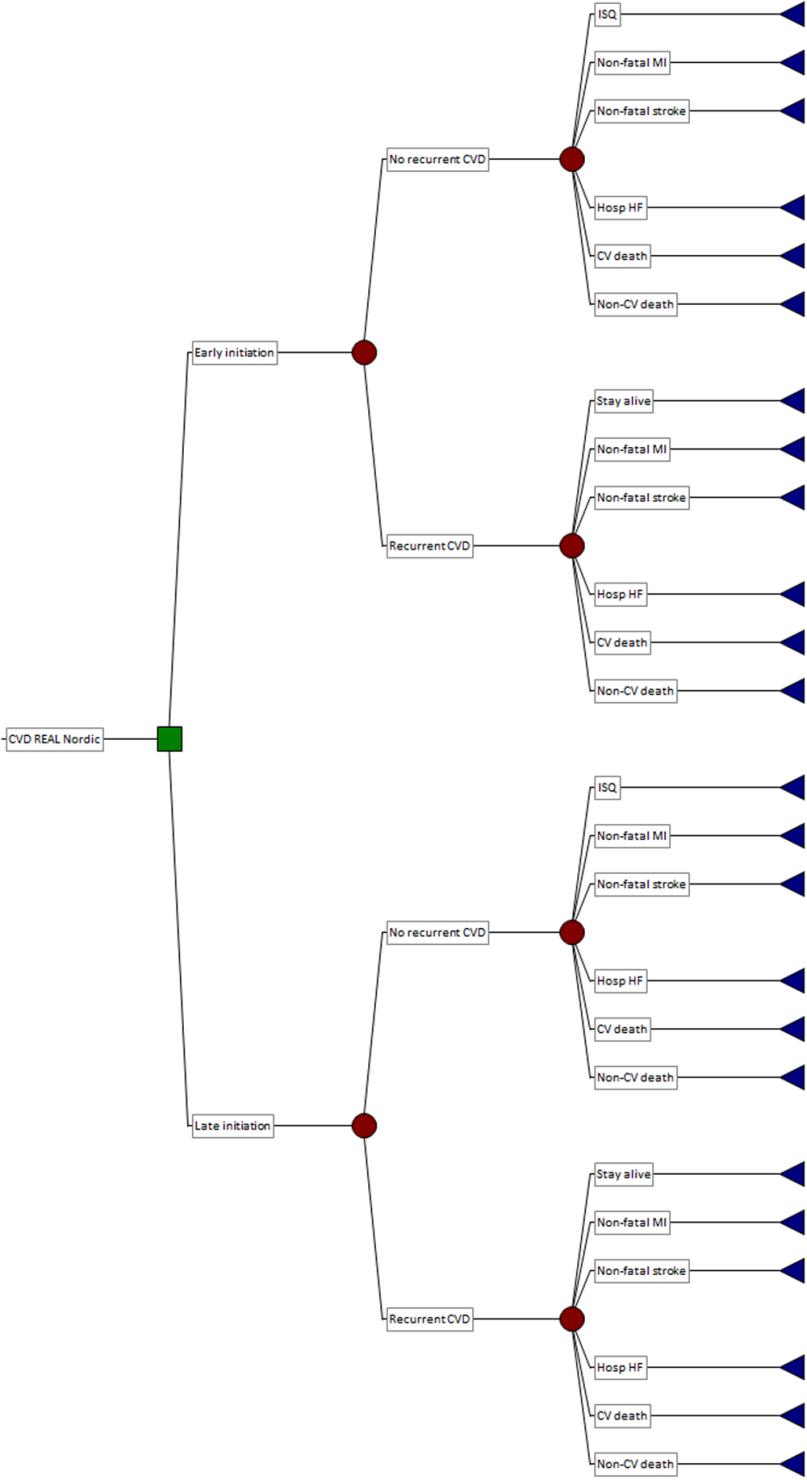
Decision analytic Markov model.

A decision analytic transition Markov model with one-year cycles was developed to compare the health and economic effects of first-line (initial) versus delayed use of combination dapagliflozin and metformin in patients with T2DM. The model structure is illustrated in Fig. 1. The model population (with an arbitrary 1000 subjects) was profiled on participants in the CVD-REAL Nordic study. We referred to this study as it reported more comprehensive data on a broad range of cardiovascular outcomes than the original study, CVD-REAL^21^, at the time we undertook our analysis. In CVD-REAL Nordic, HF and death represented 40% of all cardiovascular events.

The model had three health states: ‘Without established CVD (comprising previous myocardial infarction [MI], HF and/or stroke)’, ‘With established CVD’ and ‘Dead’. Seventy five percent of subjects began the simulation in the health state ‘Without established CVD’, and the remainder in the health state ‘With established CVD’, as per the baseline prevalence of CVD in the CVD-Real Nordic population. In each of the two living health states, subjects could progress through four possible transition states in any cycle: (i) ‘No MI, HF or stroke, stay alive’; (ii) ‘Develop non-fatal MI’ (iii) ‘Develop non-fatal stroke’; (iv) ‘Hospitalisation for HF’; (v) ‘Die from a cardiovascular cause’; and (vi) ‘Die from a non-cardiovascular cause’.

In the base case analysis, first-line combination therapy was defined as commencement of treatment with dapagliflozin 10 mg/day and metformin 1 g/day. In the comparator (delayed use) group, subjects were started with metformin 1 g alone, and subsequently switched to the combination of dapagliflozin 10 mg/day and metformin 1 g/day, according to the trend observed in the ‘10% PBS dataset’. Other than the timing of SGLT2i use, treatment with other pharmacological agents and devices were assumed to be the same in both groups over the entire time horizon. The time horizon of the model was 20 years and the costs were calculated based on 2018 Australian dollars (AUD). A discount rate of 5% per annum was applied to all costs, years of life lived and quality adjusted life years (QALYs) lived, as per Australian guidelines^22^. The outcome of interest was the incremental cost-effectiveness ratios (ICERs) in terms of AUD per years of life saved (YoLS) and AUD per QALY gained.

We also re-analysed the model using pooled estimates from Zelniker’s meta-analysis^17^, (which was published after the original submission of present manuscript), to check on the robustness of our results. The proportion of patients with established CVD and without established CVD in this study were 60.2% and 39.8%, respectively.

### Transition probabilities

The incidence rates of MI, stroke, hospitalisation for HF and death were based on observed data in CVD-REAL Nordic. However, these were not reported separately according to the presence or absence of established CVD (i.e. primary or secondary prevention). Therefore, data were also drawn from the Reduction of Atherothrombosis for Continued Health (REACH) Registry, an international, prospective cohort of 68,236 patients enrolled from 44 countries with either established atherosclerotic arterial disease or high risk of atherothrombosis^23^. Annualised incidences according to presence or absence of established CVD in patients were estimated by weighting the relative risks of events between patients with and without established CVD in REACH against incidence rates from CVD-REAL Nordic. These derived transition probabilities were applied in the first model cycle. In subsequent cycles, we applied age-related trends extracted from 2014 mortality data provided in the Australian Institute of Health and Welfare’s (AIHW) General Record of Incidence of Mortality (GRIM)^24^.

Subjects in the comparator group who switched to combination dapagliflozin and metformin assumed the cycle-specific transition probabilities of subjects in the first-line combination therapy group. Key data inputs for the model are summarised in Table 1.

**Table 1:** Key model inputs.

Parameter	Base case values	Ranges used in one-way sensitivity analyses		Distribution in PSA	References
***Transition pattern***					
Base case	Obtained from PBS data, switch by following y = 0.008e^0.2451x^			N/A	
Introduction of dapagliflozin in comparator arm			50% in Year 2, 30% in Year 3 and 20% in Year 4		
			20% per cycle from Years 4 to 8		
			10% per year from Years 1 to 10		
			20% per year from Years 1 to 20		
			None		
***Baseline event rates, % per year***	***Late initiation***	***Early initiation***			
Without established CVD			N/A	Lognormal	^14, 23^
Non-fatal MI	0.78%	0.68%			
Non-fatal stroke	0.60%	0.53%			
Hospitalisation for HF	1.12%	0.78%			
CV deaths	1.53%	0.20%			
Non-CV deaths	1.46%	0.73%			
With established CVD					
Non-fatal MI	1.25%	1.09%			
Non-fatal stroke	1.40%	1.22%			
Hospitalisation for HF	2.24%	1.57%			
CV deaths	3.76%	0.49%			
Non-CV deaths	1.86%	0.93%			
***Utilities***					
No recurrent CVD	0.778		0.775 to 0.783	Beta	^30^
Recurrent CVD	0.751		0.739 to 0.763		
***Annual background costs***					
No recurrent CVD	AUD $4,712		±50%	Lognormal	^25^
Recurrent CVD	AUD $8,727		±50%		
***Acute disease costs***					
Non-fatal MI	AUD $8,638		±50%	Lognormal	^26, 27^
Non-fatal stroke	AUD $6,988				
Hospitalisation for HF	AUD $6,168				
CV deaths	AUD $1,969		±50%		
Non-CV deaths	AUD $1,969		±50%		
***Fixed costs, per year***					
Metformin 1 g	AUD $65				
Dapagliflozin 10 mg/day plus metformin 1 g	AUD $783				

### Costs and utilities

Annual disease costs per person living with diabetes without any macrovascular complications and with macrovascular complications were assumed to be AUD $4,712 and $8,727, respectively^25^. The costs of a non-fatal MI, stroke, hospitalisation for HF and cardiovascular deaths were derived from the latest available data from Australian Refined Diagnosis Related Groups (AR-DRGs)(2014–15)^26,27^. These were AUD $8,638, $6,988, $6,168 and $3,938, respectively. We assumed that only half of all cardiovascular deaths would be hospitalised. Hence, the cost of cardiovascular deaths was AUD $1,969. Due to a lack of data regarding the costs of non-cardiovascular deaths, we assumed that these were equal to the costs of cardiovascular deaths, as has been done before^28^.

The costs of dapagliflozin and metformin were those met by the PBS, which already funds these drugs for patients with T2DM^29^. The cost of combination dapagliflozin and metformin per day was AUD $2.14, equating to an annual cost of $783. The cost of metformin per day was AUD $0.28, equating to an annual cost of $100.

Utility values according to different health states, were obtained from published literature^30^, and are summarised in Table 1.

### Sensitivity analyses

We quantified the uncertainty of the model outputs by performing one-way sensitivity analyses with variation to key data inputs one at a time. The values of the key data inputs were varied deterministically across ranges specified in Table 1.

Probabilistic sensitivity analyses (PSA) were also performed allowing all input parameters to vary stochastically. A Monte Carlo simulation^31^ with 10,000 iterations was performed by including the utilities (using beta distributions), transition probabilities (using lognormal distributions), event and annual disease costs (using lognormal distributions)^32^. Costs of drug treatment were not varied because these are fixed in the PBS. Table 1 summarises the uncertainty distributions used for the key model inputs.

Microsoft Excel (Microsoft Corporation, Redmond, WA) and @Risk version 7.5 (Palisade Corporation, New York, NY) were used to create and analyse the model.

## Results

### Prescribing of metformin and SGLT2 inhibitors

A total of 2,457,470 scripts of glucose lowering drugs were dispensed via the PBS between 1 January 2013 and 31 December 2016, involving 73,358 patients. Of this cohort, 66.2% were initiated with metformin monotherapy. Among those who were initiated with metformin monotherapy and later had a SGLT2 inhibitor added as the second agent (N = 2,331), 17%, 18%, 35% and 30% had SGLT2 inhibitors added in Years 1, 2, 3 and 4, respectively. In the modelled analysis, these were the proportions of (surviving) subjects in the comparator arm that switched to combination therapy in the first four cycles. The median time taken for a SGLT2 inhibitor to be added as the second glucose lowering drug in this arm was 2.5 years.

### Base case analysis

The model projected that over the 20-year time horizon, the 1000 subjects in the first-line combination treatment group would experience 428 episodes of non-fatal MI, 427 non-fatal stroke, 573 hospitalisations for HF, 167 cardiovascular deaths, 348 non-cardiovascular deaths and 515 all-cause deaths. In contrast, the 1000 subjects in the delayed combination treatment (comparator) group would experience 297 episodes of non-fatal MI, 292 non-fatal stroke, 606 hospitalisation for HF, 395 cardiovascular deaths, 337 non-cardiovascular deaths and 732 all-cause deaths. The differences resulted in numbers needed to treat (NNT) over a 20-year period of −8, −7, 30, 4, −93 and 5, respectively.

On average, each subject in the first-line combination treatment group was projected to live 11.0 years (discounted) and 8.5 QALYs (discounted), while each subject in the delayed combination treatment group was projected to live 8.6 years (discounted) and 6.6 QALYs (discounted). Total discounted treatment and disease-related costs per person were AUD $8,643 and AUD $76,556 in the first-line treatment group and AUD $2,304 and AUD $59,527 in the delayed combination treatment groups, respectively. These amounted to net costs of AUD $85,199 in the first-line treatment group and AUD $61,831 in the delayed combination treatment group. These figures equated to ICERs of first-line treatment versus delayed treatment of AUD $9,535 per YoLS and AUD $12,477 per QALY gained.

First-line treatment remained cost-effective when Zelniker’s data were used in the model (Supplementary). The 1000 subjects in the first-line combination treatment group would experience 540 episodes of non-fatal MI, 338 non-fatal stroke, 294 hospitalisations for HF, 421 cardiovascular deaths, 225 non-cardiovascular deaths and 646 all-cause deaths. In contrast, the 1000 subjects in the delayed combination treatment (comparator) group would experience 547 episodes of non-fatal MI, 323 non-fatal stroke, 476 hospitalisations for HF, 436 cardiovascular deaths, 230 non-cardiovascular deaths and 667 all-cause deaths. The differences resulted in numbers needed to treat (NNT) over a 20-year period of 152, −66, 5, 66, 157 and 47, respectively.

On average, each subject in the first-line combination treatment group was projected to live 10.3 years (discounted) and 7.8 QALYs (discounted), while each subject in the delayed combination treatment group was projected to live 10.0 years (discounted) and 7.6 QALYs (discounted). Total discounted treatment and disease-related costs per person were AUD $8,070 and AUD $80,959 in the first-line treatment group and AUD $2,798 and AUD $79,574 in the delayed combination treatment groups, respectively. These amounted to net costs of AUD $89,029 in the first-line treatment group and AUD $82,373 in the delayed combination treatment group. These figures equated to ICERs of AUD $24,688 per YoLS and AUD $32,552 per QALY gained.

### One-way sensitivity analyses

The ICER did not increase above $50,000 per QALY saved with the specified variations to the event costs (outlined in Table 1). In the one-way sensitivity analyses, the results remained consistent when different rates of treatment switching were applied in the delayed combination treatment group (Table 2). The cost-effectiveness results were most sensitive to the incidence rate of events in patients with and without established CVD.

**Table 2:** Results of the one-way sensitivity analyses.

Variables	Input values	ICER (Cost per YoLS)	ICER (Cost per QALY)
*Base case*		$9,535	$12,477
Introduction of dapagliflozin in comparator arm	50% in Year 2, 30% in Year 3 and 20% in Year 4	$8,577	$11,225
	20% per cycle from Years 4 to 8	$9,265	$12,120
	10% per year from Years 1 to 10	$9,203	$12,043
	20% per year from Years 1 to 20	$8,196	$10,711
	None	$9,791	$12,826
***Annual disease costs***			
Annual disease costs for patients with no recurrent CVD [reduced by 50%]	$2,356	$8,387	$10,976
Annual disease costs for patients with no recurrent CVD [increased by 50%]	$7,068	$10,682	$13,979
Annual disease costs for patients with recurrent CVD [reduced by 50%]	$4,364	$7,296	$9,548
Annual disease costs for patients with recurrent CVD [increased by 50%]	$13,091	$11,773	$15,406
***Acute costs***			
Cost of non-fatal MI [reduced by 50%]	$4,319	$9,424	$12,333
Cost of non-fatal MI [increased by 50%]	$12,957	$9,645	$12,622
Cost of non-fatal stroke [reduced by 50%]	$3,494	$9,441	$12,355
Cost of non-fatal stroke [increased by 50%]	$10,482	$9,628	$12,599
Cost of hospitalisation for HF [reduced by 50%]	$3,084	$9,568	$12,521
Cost of hospitalisation for HF [increased by 50%]	$9,251	$9,501	$12,434
Cost of CV deaths [reduced by 50%]	$984	$9,608	$12,598
Cost of CV deaths [increased by 50%]	$2,953	$9,461	$12,341
Cost of non-CV deaths [reduced by 50%]	$984	$9,542	$12,589
Cost of non-CV deaths [increased by 50%]	$2,953	$9,527	$12,367
***Utilities***			
Utility of patients with no recurrent CVD [reduced by 50%]	0.775	$9,535	$12,501
Utility of patients with no recurrent CVD [increased by 50%]	0.783	$9,535	$12,438
Utility of patients with recurrent CVD [reduced by 50%]	0.739	$9,535	$12,579
Utility of patients with recurrent CVD [increased by 50%]	0.763	$9,535	$12,378

Note: In the delayed combination treatment group, the proportion of patients switched to combination therapy in each cycle were varied deterministically. Abbreviation: CVD, cardiovascular disease; ICER, incremental cost-effectiveness ratio; MI, myocardial infarction; YoLS, years of life saved; QALY, quality-adjusted-life-years.

### Probabilistic sensitivity analysis

**Figure 2:**
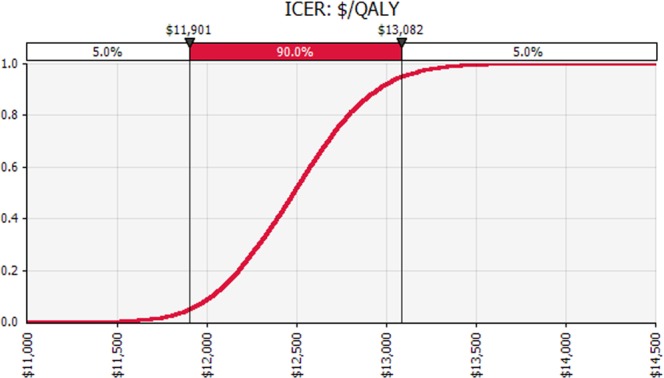
Cost-effectiveness acceptability curves illustrating the probability of first line use of combination dapagliflozin and metformin being cost-effective (10,000 simulations).

**Figure 3:**
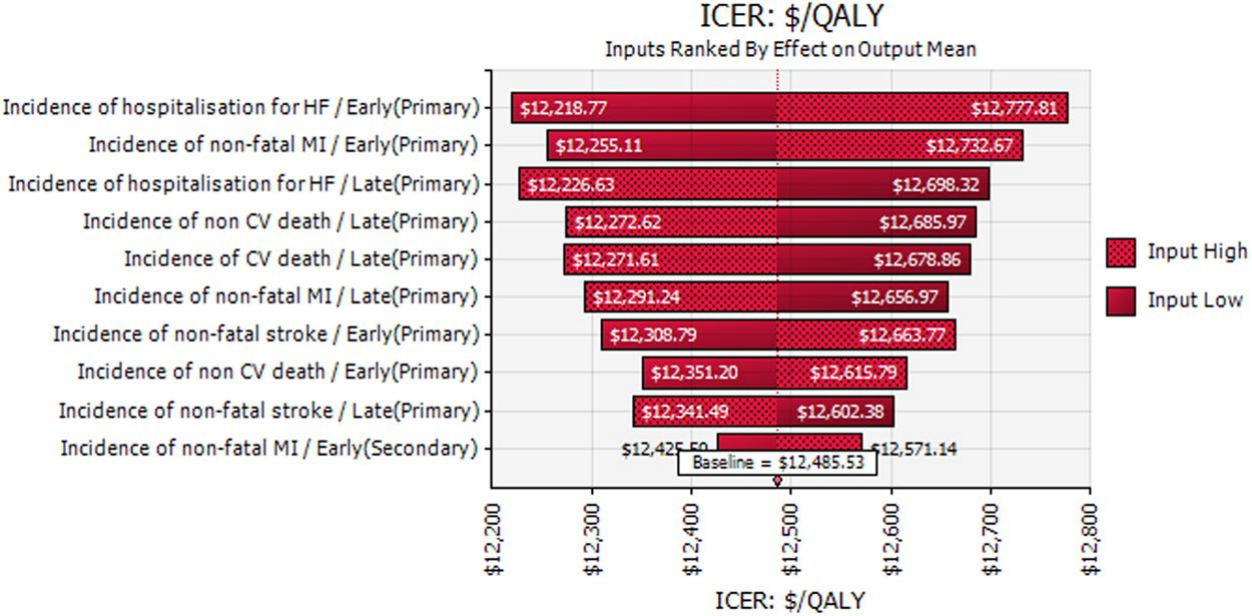
Tornado diagram illustrating the effect of variations to key input data on the cost-effectiveness of first line use of combination dapagliflozin and metformin (10,000 simulations). Note: Early (Primary); first line combination treatment for primary prevention (i.e. patients without established CVD), Late (Primary); delayed combination treatment for primary prevention (i.e. patients without established CVD), Late (Secondary); delayed combination treatment for secondary prevention (i.e. patients with established CVD).

The results of the PSA are presented as a cost-effectiveness acceptability curve in Fig. 2 and a ‘tornado diagram’ in Fig. 3. Monte-Carlo simulation showed that first-line treatment with combination dapagliflozin and metformin was cost-effective in 100% of 10,000 iterations at a willingness to pay threshold of AUD $50,000 per QALY saved. When the threshold was set at AUD $13,000 per QALY saved, there was a 92.1% of probability that first-line treatment with dapagliflozin and metformin would be cost-effective.

## Discussion

Our findings highlight the potential health and economic gains from first-line use of combination dapagliflozin and metformin among patients with T2DM. We found that first-line treatment with combination treatment is likely to be cost-effective compared to delayed use of the two drugs in Australia. Additional sensitivity analysis using the pooled estimates from Zelniker’s study did not change the conclusions.

To our knowledge, our study is the first to assess the cost-effectiveness of first-line combination treatment with a SGLT2 inhibitor and metformin compared to ‘delayed’ combination treatment. Combination metformin and dapagliflozin has been previously found to be more cost-effective than the combination of metformin and other glucose lowering drugs for reducing diabetes-related complications. Dapagliflozin in combination with metformin was reported to be more cost-effective than a sulphonylurea or dipeptidyl-peptidase-4 inhibitor (DPP4i), when added to metformin in T2DM patients inadequately controlled on metformin alone in Greece^33^. The cost per QALY gained with dapagliflozin and metformin ranged from €4769 to €7944 compared with metformin and sulphonylurea^34^. In addition, dapagliflozin in combination with metformin was more cost-effective than DPP4i in combination with metformin in the UK with ICER of £6761 per QALY gained^35^.

Data on cost-effectiveness of other SGLT2 inhibitors are consistent with those of dapagliflozin. Nguyen *et al*. demonstrated that empagliflozin was cost-effective compared to standard therapy in T2DM patients at high cardiovascular risk at a willingness-to-pay threshold of USD$100,000 per QALY gained, and remained cost-effective in 96% of 10,000 iterations^36^. Likewise, canagliflozin 300 mg and 100 mg were cost-effective compared to sitagliptin 100 mg in T2DM patients who had inadequate glucose control with metformin, with ICERs of USD$834 and USD$9,590, respectively^37^.

Considerable debate exists over adopting more intensive treatment strategies early in the course of T2DM. Major barriers to this approach include increased tendency for adverse events and costs. Other factors include clinical inertia, fear of hypoglycaemia, insulin aversion, the need for self-monitoring of blood glucose and non-adherence to prescribed medications^38^. However, findings from subsequent meta-analyses of randomised clinical trials indicate that intensive treatment significantly reduces major coronary events compared to standard glycaemic control^39–41^. Importantly, these data were generated before the emergence of the SGLT2 inhibitors and incretin modulators.

The risk of cardiovascular death is significantly increased when HbA1c levels exceed 7% in patients with T2DM^42,43^. For each 1% increase in HbA1c, the risk of cardiovascular death in patients with T2DM increases by 17% (relative risk [RR]; 1.17, 95% CI; 1.12 to 1.23)^44^. Initiating metformin monotherapy as per recommended by contemporary guidelines may reduce fasting plasma glucose levels by 2 mmol/L (95% CI, −2.4 to −1.7) and HbA1c by 0.9% (95% CI, −1.1% to −0.7%) compared with placebo in a meta-analysis of randomised control trials^45^. However, attainment of long-term maintenance of glycaemic control with any oral glucose lowering drug monotherapy is difficult in most patients, potentially due to progressive reduction in the ability to produce insulin and poor response to therapy^46,47^. Early treatment intensification confers better glycaemic control compared to late intensification (odds ratio [OR]; 1.36, 95% CI; 1.09 to 1.72], with greater effects observed among patients with higher HbA1c levels (OR; 1.53, 95% CI; 1.08 to 2.19 and OR; 2.63, 95% CI; 1.40 to 5.27, among patients with HbA1c ≥ 8% and ≥9% at baseline, respectively)^48^.

SGLT2 inhibitors act in the kidney by inhibiting SGLT2-mediated glucose reabsorption in the proximal tubules^49^. Their effects are glucose-dependent, thus leading to lower risk of hypoglycaemia, albeit higher risk of genitourinary infections. The risks of adverse events were lower with SGLT2 inhibitors than with other glucose lowering drugs in the CVD-REAL Nordic study^14^. Furthermore, the risks of confirmed hypoglycaemia, acute kidney failure and hyperkalaemia were similar between SGLT2 inhibitors and placebo in the EMPA-REG OUTCOME and CANVAS trials^12,13^. Of note, canagliflozin was associated with higher risk of toe or metatarsal amputation in CANVAS (HR, 1.97; 95% CI, 1.41 to 2.75)^12^. However, there was no observed differences in the risk of below-knee lower extremity amputation between canagliflozin, other SGLT2 inhibitors (empagliflozin and dapagliflozin) and all non-SGLT2 inhibitors in a meta-analysis of four large observational databases (OBSERVE-4D), regardless of the presence or absence of established CVD [on-treatment analysis, HR, 0.75; 95% CI, 0.40 to 1.41; and intent-to-treat analysis, HR, 1.01; 95% CI, 0.93 to 1.10]^50^. To date, SGLT2 inhibitors comprise the only class of oral antihyperglycaemic drugs with evidence of direct cardiovascular benefits. Therefore, the concept of first-line initiation of dapagliflozin and metformin is appealing on this basis.

Our analysis has several limitations that warrant mention. First, our modelled analysis adopted the perspective of the Australian healthcare system and therefore the results may not be directly applicable to other settings. Secondly, treatment effects were primarily derived from an observational study and analysed under the assumption that the benefits observed in CVD-REAL Nordic would be observed in an Australian population with T2DM. However, the benefits were only applied in the first year of the modelled analysis. From the second year onwards, only age-related increases in disease and death risks were assumed, and these were the same for both the initial and delayed groups. Another limitation stemmed from the assumptions that non-fatal MI, stroke and hospitalisation for HF only occurred once per cycle (year) and that 50% of deaths occurred in hospital to simplify calculations and due to lack of data. Such assumptions may not hold true in real life settings, but they were conservative, and hence would have led to under-estimation of the true cost-effectiveness of initial combination treatment. Finally, we did not model changes in diabetes-related complications (such as diabetic nephropathy and dialysis), adverse events and adherence over time, each of which may have affected our results in an unpredictable fashion depending on the extent and differences between the early and late initiation of dapagliflozin and metformin. Again, any potential inaccuracies with these input parameters was unlikely to have changed the conclusion of our study that up-front use of combination therapy is cost-effective.

## Conclusions

In summary, compared to initial metformin monotherapy followed by gradual addition of dapagliflozin, first-line use of combination dapagliflozin and metformin is likely to be a cost-effective approach in the management of Australians with T2DM.

## Supplementary information

Supplementary

## Data Availability

The datasets used and/or analyzed during the current study are available from the corresponding author on reasonable request.

## References

[1] Chen L, Magliano DJ, Zimmet PZ (2011). The worldwide epidemiology of type 2 diabetes mellitus - present and future perspectives. Nat Rev Endocrinol.

[2] Levelt E, Gulsin G, Neubauer S, McCann GP (2018). Diabetic cardiomyopathy: pathophysiology and potential metabolic interventions state of the art review. Eur J Endocrinol.

[3] Fox CS (2004). Trends in cardiovascular complications of diabetes. JAMA.

[4] Herman WH, Cohen RM (2012). Racial and ethnic differences in the relationship between HbA1c and blood glucose: Implications for the diagnosis of diabetes. J Clin Endocrinol Metab.

[5] Adams AS (2008). Medication adherence and racial differences in A1C control. Diabetes Care.

[6] Kirk JK (2008). Disparities in A1C levels between Hispanic and non-Hispanic white adults with diabetes: a meta-analysis. Diabetes Care.

[7] Blonde L (2017). Gaps and barriers in the control of blood glucose in people with type 2 diabetes. Diab Vasc Dis Res.

[8] American Diabetes Association (2018). Standards of medical care in diabetes - 2018. Diabetes Care.

[9] Garber AJ (2018). Consensus statement by the American Association of Clinical Endocrinologists and American College of Endocrinology on the comprehensive type 2 diabetes management algorithm - 2018 executive summary. Endocr Pract.

[10] Gunton JE, Cheung NW, Davis TME, Zoungas S, Colagiuri S (2014). A new blood glucose management algorithm for type 2 diabetes: A position statement of the Australian Diabetes Society. MJA.

[11] Gunton, J. E., Cheung, N. W., Davis, T. M. E., Colagiuri, S. & Zoungas, S. *A new blood glucose management algorithm for type 2 diabetes: A position statement of the Australian Diabetes Society. Updated December* 2018*. Available at*, https://diabetessociety.com.au/position-statements.asp *Accessed on* (June 12, 2018). 10.5694/mja14.01187 25495309

[12] Neal B (2017). Canagliflozin and cardiovascular and renal events in type 2 diabetes. N Engl J Med.

[13] Zinman B (2015). Empagliflozin, cardiovascular outcomes, and mortality in type 2 diabetes. N Engl J Med.

[14] Birkeland KI (2017). Cardiovascular mortality and morbidity in patients with type 2 diabetes following initiation of sodium-glucose co-transporter-2 inhibitors versus other glucose-lowering drugs (CVD-REAL Nordic): a multinational observational analysis. Lancet Diabetes Endocrinol.

[15] Wiviott SD (2019). Dapagliflozin and cardiovascular outcomes in type 2 diabetes. N Engl J Med.

[16] Kosiborod M (2018). Cardiovascular events associated with SGLT-2 inhibitors versus other glucose-lowering drugs: The CVD-REAL 2 Study. J Am Coll Cardiol.

[17] Zelniker TA (2019). SGLT2 inhibitors for primary and secondary prevention of cardiovascular and renal outcomes in type 2 diabetes: a systematic review and meta-analysis of cardiovascular outcome trials. Lancet.

[18] Mellish L (2015). The Australian Pharmaceutical Benefits Scheme data collection: a practical guide for researchers. BMC Res Notes.

[19] Ofori-Asenso, R. *et al*. Patterns of statin use and long-term adherence and persistence among older adults with diabetes. *J Diabetes*, 10.1111/1753-0407.12769 [Epub ahead of print] (2018). 10.1111/1753-0407.12769 29658177

[20] Zhao Y, Condon J, Lawton P, He V, Cadilhac DA (2016). Lifetime direct costs of stroke for indigenous patients adjusted for comorbidities. Neurology.

[21] Kosiborod M (2017). Lower risk of heart failure and death in patients initiated on sodium-glucose cotransporter-2 inhibitors versus other glucose-lowering drugs: The CVD-REAL Study (Comparative effectiveness of cardiovascular outcomes in new users of sodium-glucose cotransporter-2 inhibitors). Circulation.

[22] Department of Health. Guidelines for preparing a submission to the Pharmaceutical Benefits Advisory Committee (Version 5.0). Available at, https://www.ispor.org/PEguidelines/countrydet.asp?c=1&t=2 Accessed (Nov 24, 2017).

[23] Steg PG (2007). One-year cardiovascular event rates in outpatients with atherothrombosis. JAMA.

[24] Australian Institute of Health and Welfare (AIHW). National GRIM books. Australian Institute of Health and Welfare, http://www.aihw.gov.au/reports/life-expectancy-death/grim-books/contents/grim-books Accessed (Nov 2017).

[25] Lee CMY (2013). The cost of diabetes in adults in Australia. Diabetes Res Clin Pract.

[26] Independent Hospital Pricing Authority (IHPA). *National Hospital Cost Data Collection, Public Hospitals Cost Report, Round 19 (Financial year 2014–15). Available at*, https://www.ihpa.gov.au/publications/national-hospital-cost-data-collection-public-hospitals-cost-report-round-19-financial *Accessed* (December 6, 2017).

[27] Independent Hospital Pricing Authority. NHCDC Cost Report, Round 19 (Financial year 2014–15) appendix (2014).

[28] Ademi Z, Pasupathi K, Krum H, Liew D (2014). Cost Effectiveness of eplerenone in patients with chronic heart failure. Am J Cardiovasc Drugs.

[29] Pharmaceutical Benefits Scheme (PBS). *Available at*, https://www.pbs.gov.au/pbs/home *Accessed at* (Nov 24, 2017).

[30] Briggs AH (2017). Health-related quality-of-life implications of cardiovascular events in individuals with type 2 diabetes mellitus: A subanalysis from the Saxagliptin Assessment of Vascular Outcomes Recorded in Patients with Diabetes Mellitus (SAVOR)-TIMI 53 trial. Diabetes Res Clin Pract.

[31] Briggs AH, Gray AM (1999). Handling uncertainty in economic evaluations of healthcare interventions. BMJ.

[32] Briggs AH (2012). Model parameter estimation and uncertainty: A report of the ISPOR-SMDM Modeling Good Research Practices Task Force-6. Value Health.

[33] Tzanetakos C, Tentolouris N, Kourlaba G, Maniadakis N (2016). Cost-effectiveness of dapagliflozin as add-on to metformin for the treatment of type 2 diabetes mellitus in Greece. Clin Drug Investig.

[34] Sabale U, Ekman M, Granström O, Bergenheim K, McEwan P (2015). Cost-effectiveness of dapagliflozin (Forxiga®) added to metformin compared with sulfonylurea added to metformin in type 2 diabetes in the Nordic countries. Prim Care Diabetes.

[35] Charokopou M (2015). Cost-effectiveness of dapagliflozin versus DPP-4 inhibitors as an add-on to metformin in the treatment of type 2 diabetes mellitus from a UK Healthcare System Perspective. BMC Health Serv Res.

[36] Nguyen E, Coleman CI, Nair S, Weeda ER (2018). Cost-utility of empagliflozin in patients with type 2 diabetes at high cardiovascular risk. J Diabetes Complications.

[37] Neslusan C (2015). Cost-effectiveness of canagliflozin versus sitagliptin as add-on to metformin in patients with type 2 diabetes mellitus in Mexico. Value Health Reg Issues.

[38] Owens DR, Monnier L, Barnett AH (2017). Future challenges and therapeutic opportunities in type 2 diabetes: Changing the paradigm of current therapy. Diabetes Obes Metab.

[39] Mannucci E, Monami M, Lamanna C, Gori F, Marchionni N (2009). Prevention of cardiovascular disease through glycemic control in type 2 diabetes: A meta-analysis of randomized clinical trials. Nutr Metab Cardiovasc Dis.

[40] Ray KK (2009). Effect of intensive control of glucose on cardiovascular outcomes and death in patients with diabetes mellitus: a meta-analysis of randomised controlled trials. Lancet.

[41] Turnbull FM (2009). Intensive glucose control and macrovascular outcomes in type 2 diabetes. Diabetologia.

[42] Cavero-Redondo I, Peleteiro B, Álvarez-Bueno C, Rodriguez-Artalejo F, Martínez-Vizcaíno V (2017). Glycated haemoglobin A1c as a risk factor of cardiovascular outcomes and all-cause mortality in diabetic and nondiabetic populations: a systematic review and meta-analysis. BMJ Open.

[43] Zoungas S (2012). Association of HbA1c levels with vascular complications and death in patients with type 2 diabetes: evidence of glycaemic thresholds. Diabetologia.

[44] Zhang Y, Hu G, Yuan Z, Chen L (2012). Glycosylated hemoglobin in relationship to cardiovascular outcomes and death in patients with type 2 diabetes: A systematic review and meta-analysis. PLoS ONE.

[45] Johansen K (1999). Efficacy of metformin in the treatment of NIDDM. Diabetes Care.

[46] Turner RC, Cull CA, Frighi V, Holman RR (1999). & for the UK Prospective Diabetes Study (UKPDS) Group. Glycemic control with diet, sulfonylurea, metformin, or insulin in patients with type 2 diabetes mellitus: Progressive requirement for multiple therapies (UKPDS 49). JAMA.

[47] Kahn SE (2006). Glycemic durability of rosiglitazone, metformin, or glyburide monotherapy. N Engl J Med.

[48] Rajpathak SN, Rajgopalan S, Engel SS (2014). Impact of time to treatment intensification on glycemic goal attainment among patients with type 2 diabetes failing metformin monotherapy. J Diabetes Complications.

[49] Marx N, McGuire DK (2016). Sodium-glucose cotransporter-2 inhibition for the reduction of cardiovascular events in high-risk patients with diabetes mellitus. Eur Heart J.

[50] Ryan, P. B. *et al*. Comparative effectiveness of canagliflozin, SGLT2 inhibitors and non-SGLT2 inhibitors on the risk of hospitalization for heart failure and amputation in patients with type 2 diabetes mellitus: A real-world meta-analysis of 4 observational databases (OBSERVE-4D). *Diabetes Obes Metab*, In press, 10.1111/dom.13424 [Epub ahead of print] (2018). 10.1111/dom.13424 PMC6220807 29938883

