# Supplementary material for: Cost-effectiveness of first-line versus delayed use of combination dapagliflozin and metformin in patients with type 2 diabetes
Source: Sci Rep. 2019 Mar 1;9:3256. doi: 10.1038/s41598-019-40191-8 (PMC6397228; doi:10.1038/s41598-019-40191-8)

Cost-effectiveness of first-line versus delayed use of combination dapagliflozin and metformin in patients with type 2 diabetes

(Running title: CEA of first-line dapagliflozin and metformin)

Ken Lee Chin, PhD[1,2], Richard Ofori-Asenso, MSc[1], Si Si, PhD[1], Thomas R. Hird, PhD[1,3], Dianna J. Magliano, PhD[3,4], Sophia Zoungas, MBBS, PhD[4,5], Danny Liew, MBBS, PhD[1]

[1]CCRE Therapeutics, Department of Epidemiology and Preventive Medicine, Monash University, Melbourne, Australia

[2]Melbourne Medical School, The University of Melbourne, Parkville, Australia

[3]Baker Heart and Diabetes Institute, Melbourne Australia

[4]Department of Epidemiology and Preventive Medicine, School of Public Health and Preventive Medicine, Monash University, Melbourne, Australia.

[5]The George Institute for Global Health, Sydney, Australia

Corresponding Author:
Professor Danny Liew
Centre of Cardiovascular Research & Education (CCRE) in Therapeutics
Department of Epidemiology & Preventive Medicine
Monash University/Alfred Hospital

Commercial Road

Melbourne Vic 3004 Australia

Tel: +61 3 9903 0759

Fax: +61 3 9903 0556

Email: danny.liew@monash.edu

Email list:

Ken Lee Chin	ken.chin@unimelb.edu.au
Richard Ofori-Asenso	richard.ofori-asenso@monash.edu
Si Si	si.si@monash.edu
Thomas R. Hird	tom.hird@monash.edu
Dianna J. Magliano	dianna.magliano@baker.edu.au
Sophia Zoungas	sophia.zoungas@monash.edu
Danny Liew	danny.liew@monash.edu

Supplement

Table S1. Sensitivity analysis performed using pooled estimates from Zelniker's meta-analysis.

	**Acute events**										
	MI	Stroke	Hosp HF	CV death	Non CV death	Total death	Discounted YLL	Discounted QALYs	Costs of treatment and disease	Incremental costs/YoLs	Incremental costs/QALYs
Late initiation	546.5	323.3	476.6	436.7	230.9	667.6	10,038.0	7,637.7	$82,373,326	$24,688	$32,552
Early initiation	539.9	338.4	294.4	421.7	224.5	646.2	10,307.7	7,841.8	$89,030,065		
DIFF	-6.6	15.1	-182.2	-15.1	-6.3	-21.4	269.6	204.5	$6,656,740		
NNT	152	-66	5	66	157	47					

Figure S1. Cost-effectiveness acceptability curves illustrating the probability of first line use of combination dapagliflozin and metformin being cost-effective (10,000 simulations) - using pooled estimates from Zelniker's meta-analysis.

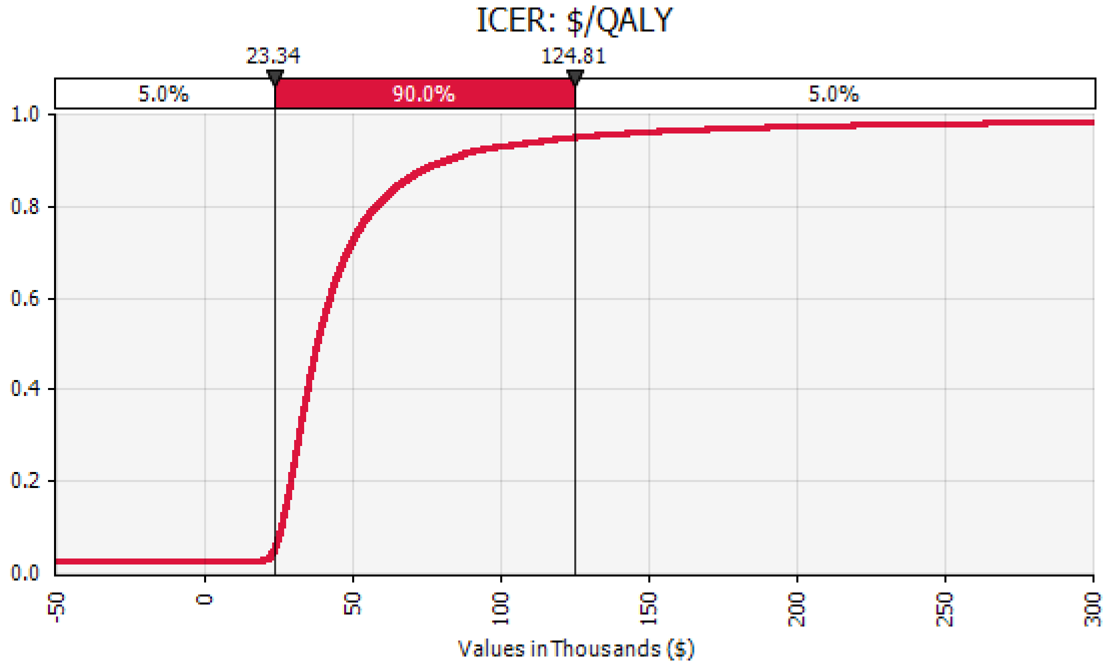

Figure S2. Tornado diagram illustrating the effect of variations to key input data on the cost-effectiveness of first line use of combination dapagliflozin and metformin (10,000 simulations) - using pooled estimates from Zelniker's meta-analysis.

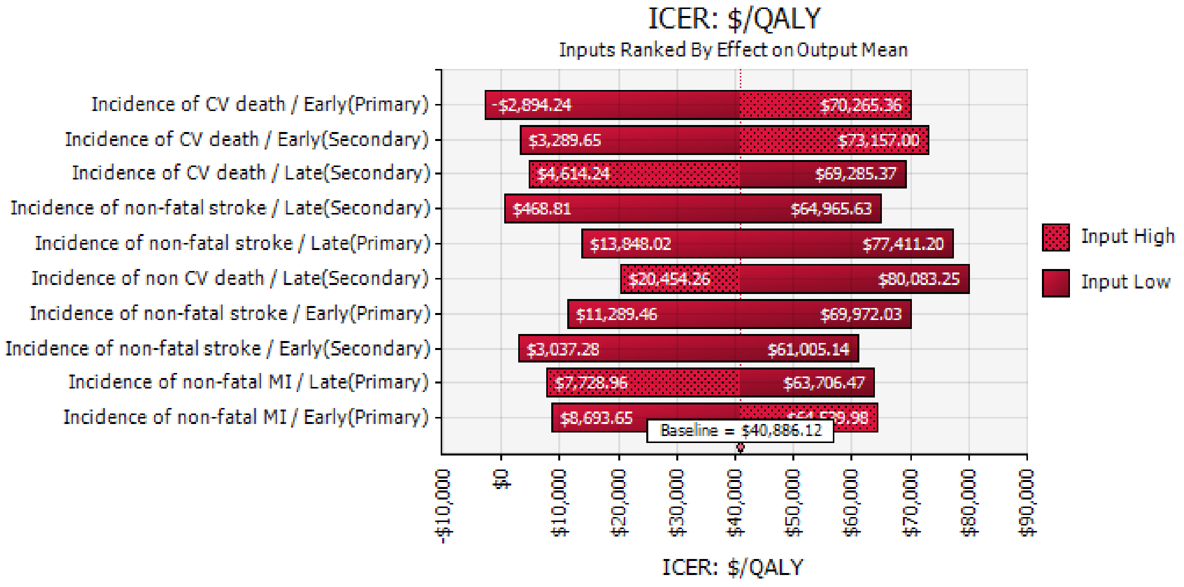

Supplement: Supplementary file 1 — Supplementary [file 41598_2019_40191_MOESM1_ESM.pdf]
